# Polio eradication in a chronic conflict setting lessons from the Republic of South Sudan, 2010-2020

**DOI:** 10.11604/pamj.supp.2022.42.1.32922

**Published:** 2022-06-09

**Authors:** Sylvester Maleghemi, Ayesheshem Ademe Tegegne, Melisachew Ferede, Bassey Enya Bassey, Godwin Ubong Akpan, Isah Mohammed Bello, Johnson Muluh Ticha, Atem Anyuon, Joy Luba Waya, Samuel Oumo Okiror, Modjirom Ndoutabe, Kibebu Kinfu Berta, Fabian Ndenzako, Pascal Mkanda, Olushayo Oluseun Olu

**Affiliations:** 1 World Health Organization, WHO Country Office, Ministerial Complex, Juba, South Sudan,; 2 World Health Organization Oyo State Office, Nigeria,; 3 World Health Organization, Regional Office for Africa, Cite de Djoue, Brazzaville, Congo,; 4 World Health Organization, Inter-Country Support Team office for East and Southern Africa, P.O. Box 5160, Harare, Zimbabwe,; 5 Ministry of Health, Ministerial Complex, Juba, South Sudan

**Keywords:** Polio eradication, acute flaccid paralysis, routine immunisation coverage, polio campaigns, wild poliovirus, wild poliovirus free certification, South Sudan

## Abstract

**Introduction:**

in 1988 the World Health Assembly set an ambitious target to eradicate Wild Polio Virus (WPV) by 2000, following the successful eradication of the smallpox virus in 1980. South Sudan and the entire African region were certified WPV free on August 25, 2020. South Sudan has maintained its WPV free status since 2010, and this paper reviewed the country’s progress, outlined lessons learned, and describes the remaining challenges in polio eradication.

**Methods:**

secondary data analysis was conducted using the Ministry of Health and WHO polio surveillance datasets, routine immunisation coverage, polio campaign data, and surveys from 2010 to 2020. Relevant technical documents and reports on polio immunisation and surveillance were also reviewed. Data analysis was conducted using EPI Info 7 software.

**Results:**

administrative routine immunisation coverage for bivalent Oral Polio Vaccine (OPV) 3rd dose declined from 77% in 2010 to 56% in 2020. In contrast, the administrative and post-campaign evaluation coverage recorded for the nationwide supplemental polio campaigns since 2011 was consistently above 85%; however, campaigns declined in number from four in 2011 to zero in 2020. Overall, 76% of notified cases of Acute Flaccid Paralysis (AFP) received three or more doses of the oral polio vaccine. The Annualized Non-AFP rate ranged between 4.0 to 5.4 per 100,000 under 15 years populations, and stool adequacy ranged from 83% to 94%.

**Conclusion:**

South Sudan’s polio-free status documentation was accepted by the ARCC in 2020, thereby enabling the African Region to be certified WPV free on August 25, 2020. However, there are concerns as the country continues to report low routine immunisation coverage and a reduction in the number of polio campaigns conducted each year. It is recommended that the country conduct high-quality nationwide supplemental polio campaigns yearly to achieve and maintain the required herd immunity. It invests in its routine immunisation program while ensuring optimal AFP surveillance performance indicators.

## Introduction

Poliomyelitis is a highly infectious disease caused by polio viruses and causes irreversible paralysis in children and adults [1]. The clinical manifestation is paralysis which occurs a few hours or days after contracting the virus. However, most polio cases are asymptomatic, which helps sustain its transmission through the faecal-oral route, with communities with poor hygiene and sanitation being at high risk [2,3]. The 41st World Health Assembly (WHA) held in 1988 adopted a resolution to eradicate polio globally [4]. Subsequently, the Global Polio Eradication Initiative (GPEI), a public-private partnership, was launched and tasked with ensuring support to all countries to eradicate the disease [5-7].

The GPEI has made tremendous progress since its establishment. As of 2020, the number of paralyses caused by WPV type 1 was 140, a reduction of over 99.9% from the original 350,000 reported in 1988 before eradication efforts were implemented [8-10]. Notably, two of the three strains of WPV that cause paralysis have been eradicated, with WPV type 2 declared eradicated in September 2015 and WPV type 3 in October 2019 [11-13]. The core strategies utilised in achieving these feats are high routine immunisation coverage, provision of supplementary Oral Polio Vaccine (OPV) doses through national immunisation days, strong Acute Flaccid Paralysis (AFP) surveillance system, and “mop ups”, which are targeted polio campaigns in areas of poliovirus transmission [14]. As of December 2020, all but one of the World Health Origination (WHO) regions, the Eastern Mediterranean Regions, have been certified wild poliovirus (WPV) free, with cases reported from Pakistan and Afghanistan [15,16]. The African Region was certified WPV free in August 2020 following Nigeria’s eradication of WPV type 1 in 2016 [17].

South Sudan has also made tremendous progress in the eradication of polio. The last indigenous wild polio virus type 1 case was reported in 2001 in Pariang County, Unity State [18]. Though indigenous wild poliovirus was interrupted in 2001, the country experienced two imported outbreaks of WPV in 2004-2005 and 2008-2009 from Nigeria via Sudan. The country’s claim of being WPV free was accepted in June 2020. However, Vaccine-Derived Polio Virus (VDPV) outbreaks continue to be recorded in the country, with the most recent outbreak declared on 18 September 2020 by the Ministry of Health (MOH) in South Sudan [19].

Polio eradication in the chronic conflict setting of South Sudan faced several challenges, including difficult access due to insecurity and terrain, disrupted health systems, destruction and looting of health facilities, and poor infrastructure. The country continues to battle with a humanitarian crisis due to the cumulative effects of years of conflict, which have destroyed people’s livelihoods. As of August 2020, the humanitarian situation report noted that 7.5 million people need humanitarian assistance, with 1.6 million internally displaced and another 2.26 million living as refugees in neighboring countries [20] with a weak health system largely dependent on donors and implementing partners. Surmounting these challenges required dedicated and trained personnel with innovative strategies and approaches that are not documented in the literature. Scientific publications on the progress, challenges and lessons learned remain scarce. This paper, therefore, reports the path towards polio eradication in South Sudan and contributes to global lessons learned and best practices in the eradication initiative. Findings from the South Sudan context could be extrapolated to other countries in conflict and accelerate the global polio eradication efforts.

## Methods

**Study area:** South Sudan is a landlocked country located in Eastern Africa. It covers approximately 640,000km2 with a projected population of 13.3 million using a 3.0% growth rate from the census figures for 2008 and a population density of 15 per square kilometre [21]. It is divided into ten states and further subdivided into 80 Counties and over 600 Payams. It attained independence on 9th July 2011, following more than two decades of civil war, with renewed civil conflicts occurring in December 2013 and again in June 2016, along with continuous fighting, which remains ongoing in the country [22]. The conflicts have spared no states or regions; however, three states, Jonglei, Unity, and Upper Nile, known as the former conflict-affected states, have felt the brunt the most and have undermined the health system’s capacity to deliver essential health services. Still, despite the challenges, active surveillance of AFP cases in health facilities and communities continues facilitated by the partnership and huge polio workforce located at even the lowest level (Boma), which was well established in the country even before its independence.

**Study design:** we conducted a retrospective descriptive study of the polio eradication initiative in South Sudan from 2010 to 2020 through secondary analyses of quantitative data from the national polio database. Qualitative data were obtained through reviewing documents and reports on polio immunisation campaigns, AFP surveillance, and other polio eradication activities. Analysis of immunisation coverage rates included data from South Sudan District Health Information System (DHIS), WHO United Nations Children’s Fund (UNICEF) Joint Reporting Form on Immunisation (JRF), and the WHO, UNICEF Immunisation Coverage Estimates (WUENIC). The polio supplemental campaign and surveillance data were obtained from the MOH and WHO databases.

**Immunisation coverage and data:** we used routine administrative coverage data for polio immunisation collected every month from approximately 1065 health facilities in 80 counties transmitted through the DHIS system to the national MOH database. Data on polio vaccination coverage were also retrieved from reports on the immunisation surveys and WUENIC. The polio campaign data were obtained from the tally sheets used during the campaigns and collated at all levels, with the final summary shared by states to the National level. Other data sets were analysed to determine the quality and extent of the campaign, including the post-campaign evaluations (PCEs) and lot quality assurance sampling (LQAS). Two vaccines protect against the poliovirus, the Oral Polio Vaccine (OPV) and Injectable Polio Vaccine (IPV). A child is said to be fully vaccinated against the poliovirus when OPV3 and IPV are recorded on their vaccination card. Oral polio vaccine (OPV3) is the third time a child receives an oral polio vaccine, excluding the OPV birth dose and given along with the IPV at 14 weeks or later. IPV was added to the immunisation schedule in Dec 2015. The country conducts supplementary immunisation campaigns using OPV. All children under five of age are targeted and given two drops of the polio vaccines irrespective of the child’s polio vaccination status, which boosts polio herd immunity. Results are collated against set targets, with an evaluation done to determine the coverage and quality of the campaign

**Acute flaccid paralysis (AFP) surveillance:** the AFP surveillance system in South Sudan relies on health facilities and community-based reporting. The system detects, notifies, investigates, and verifies AFP cases in children under 15 years old or in any person a clinician suspects poliomyelitis. In South Sudan, it involves over 4500 personnel. It is a partnership between community members, Non-Government Organisations (NGOs), International Non-Government Organisations (INGOs), Civil Societies (CSO), the Rotary, and United Nations (UN) organisations led by the MOH South Sudan. There are two main AFP surveillance indicators, non-polio AFP (NP-AFP) rate, cases of acute flaccid paralysis not due to polio per 100,000 children less than 15 years with an NP-AFP rate of ≥2 agreed as the country’s standard, being able to detect the poliovirus, and the stool adequacy, with 80% of the stool being adequate as the standard [23,24]. Adequate stool means that the stool must be collected within 14 days of onset of the paralysis and transported to a WHO accredited lab under a good reverse cold chain [24]. Apart from the two main polio surveillance indicators, another indicator that determines the quality of the AFP surveillance system is the non-polio enterovirus rate (NPENT). This determines the ability of the laboratory to report the poliovirus or any other virus if present in the AFP stool sample with a benchmark of 10% [25].

When a person meets the community AFP case definition, the sudden weakness of any limbs, two stool samples are collected with at least a 24-hour interval between stool collection, under the appropriate reverse cold chain. South Sudan transports all AFP samples from the states to the capital, Juba, via aeroplanes. From Juba, the AFP samples are flown to the Uganda Virus Research Institute laboratory (UVRI), a WHO accredited polio laboratory, for analysis. All AFP cases have a case-based form completed, including clinical and epidemiological information. Records are stored in a centralised MS Access Database by the WHO data manager, with a copy retained at the National Public Health Laboratory Juba. Detailed case investigation (DCI) is conducted for all reported AFP cases to authenticate if it is a true AFP case by senior WHO and MOH EPI officers. Follow-up of inadequate cases, “cases investigated after 14 days of paralysis or with inadequate samples collected,” is conducted by senior officers and clinicians using detailed investigation forms entered the Open Data Kit (ODK, including its website) mobile platform and uploaded into the national database. The National Polio Expert Committee (NPEC) meets quarterly and classifies all inadequate cases while endorsing adequate cases classified by the secretariat and making applicable adjustments. Laboratory results are entered into the database for completeness as soon as received from the URVI. We analysed retrospectively the AFP database stored at the national level that was routinely collected from all states during the study period.

**Review of literature:** PubMed, Global Health, and Google Scholar databases were searched for original peer-reviewed articles describing polio eradication efforts in South Sudan published from 2002 to 2020. The following combinations were used as keywords to search for literature on polio: ‘polio’ and a combination of the following words in permutations - ‘eradication + surveillance + South Sudan’, ‘vaccination + South Sudan’. The search yielded three articles, of which none were considered relevant because the content focused on other vaccines and not the oral or injectable polio vaccines. Additionally, all available country, regional and global reports on immunisation for the period of 2002 to 2020 was reviewed. Published and unpublished reports on AFP surveillance and immunisation data reported by the South Sudan Ministry of Health were studied, along with annual progress reports on poliomyelitis eradication activities submitted to the ARCC (2014-2020) and quarterly reports from the Polio eradication committees, which were also reviewed.

**Data analysis:** the database for this study was from the DHIS, a web-based open-source software platform for reporting, analysis, and dissemination of data for all health programs in the country, which was exported to MS Excel. The WHO and UNICEF for estimating global, country by country, infant immunisation coverage (WUENIC) [26], web-based, MOH campaign database, immunisation surveys, and AFP surveillance case-based database access based, all exported to Microsoft excel for validation, cleaning, and analysis. Statistical data analysis was conducted using Epi Info statistical software (version 7; CDC, Atlanta, United States). Descriptive analyses were performed to describe the epidemiology of reported AFP cases in South Sudan, and statistics based on the WHO recommended performance indicators for AFP surveillance were generated [27]. Mapping was done to visualise surveillance performance and distribution of AFP cases by location using the ArcGIS Pro software. Results of the study are presented in the form of tables and maps. Qualitative analysis for the study used reports compiled from the Annual Polio reports, the 2019-2020 Polio free documentation, and yearly GAVI Joint Appraisals and supervisory reports. These reports were reviewed, and a strengths, weaknesses, opportunities, and threats (SWOT) analysis of the polio programme was developed based on methods from Wijingaarden [28].

**Ethical approval and consent:** the Ministry of Health approved this study. We used secondary data collected and stored at WCO and MOH. Administrative clearance for publication of this editorial was provided by the Ministry of Health of South Sudan and WHO (ePub-IP-00331505-EC) to publish the result. Moreover, the Research Ethics Review Board of the Ministry of Health provided clearance for the publication of manuscript under (MoH/RERB/D.03/2022) clearance number.

## Results

**Figure 1:**
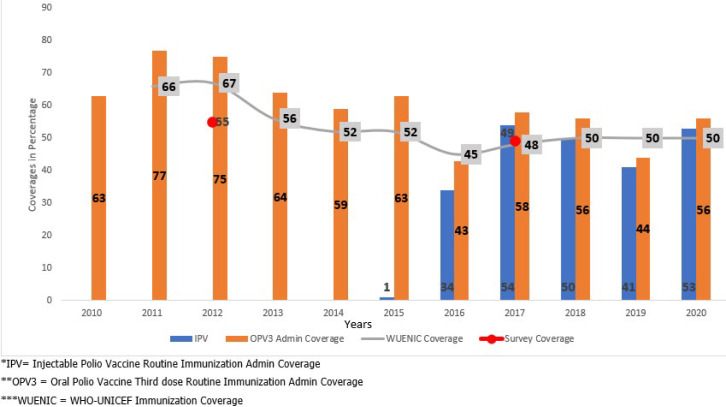
oral polio vaccine 3rd dose/ injectable polio vaccine immunisation coverage 2010-2020, South Sudan

**Routine immunisation coverage:** according to the MOH DHIS data, national administrative vaccination coverage for OPV3 ranged from 43% to 77% between 2010 and 2020. Injectable polio vaccine coverage was between 1% and 53% from 2016 to 2020 (Figure 1). A disparity exists even though both vaccines are administered simultaneously (Figure 1). The routine immunisation coverage has continued to decline from 2011, reaching its lowest level in 2016 and 2019, with administered coverage at 43% and 44%, respectively. Data from the EPI survey coverage to independently assess the EPI coverage, directly from the household conducted in the country in 2012 and 2017, estimated the OPV3 coverage to be 55% and 49%, respectively. A discrepancy of 10% was noted between the OPV3 administrative coverage and survey. The WUENIC report since 2010 showed that the OPV3 coverage has been below 60% since the conflict started in 2013 and has stagnated at 50% since 2018. The OPV3 administrative coverage is usually higher than the WUENIC report.

**Figure 2:**
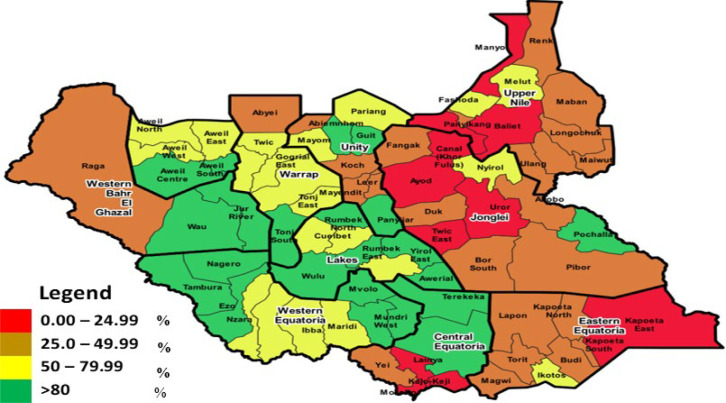
oral polio vaccine 3rd dose routine immunisation administration coverage by county, South Sudan 2020

At the state level, the OPV3 administrative coverage ranged between 12% and 124% between 2015 to 2020 (Table 1), with the three-former conflict-affected states of Jonglei, Unity, and Upper Nile accounting for states with the lowest OPV3 coverage with an 11-year average immunisation coverage of 33%, 46%, and 34% respectively. The other seven states continued to record varying and inconsistent OPV 3 coverage, with Central Equatoria and Warrap having the highest 11 years average coverage of 81% and 80%, respectively. In 2020 administrative routine immunisation data for OPV3 showed that 21 (26%) counties recorded coverage of OPV3 greater than 80%, 22 (28%) between 50-79.99%, 26 (33%) between 25-49.99% and 11 (14%) counties had OPV 3 coverage of less than 25% (Figure 2). Counties with the lowest coverage are found in Unity, Jonglei and Upper Nile states; however, two counties from Eastern Equatoria and Central Equatoria reported less than 25% in 2020.

**Table 1:** routine immunisation: oral polio vaccine 3^rd^ dose administration coverage by state 2010 - 2020, South Sudan

State	2010	2011	2012	2013	2014	2015	2016	2017	2018	2019	2020	11 years average
	%	%	%	%	%	%	%	%	%	%	%	%
Central Equatoria	70	103	109	98	117	102	60	73	64	50	50	81
Eastern Equatoria	83	65	62	62	64	76	44	53	52	29	41	57
Jonglei	44	61	67	64	8	18	15	13	21	21	33	33
Lakes	40	94	73	45	54	70	63	124	88	66	82	73
Northern Bahr El Ghazal	70	80	77	70	84	87	45	78	67	57	69	71
Unity	75	94	62	22	13	26	24	29	58	35	72	46
Upper Nile	49	69	57	56	23	12	19	19	21	21	31	34
Warrap	76	80	93	64	84	107	80	95	87	51	66	80
Western Bahr El Ghazal	96	68	81	91	88	79	46	70	57	66	97	76
Western Equatoria	45	55	62	56	83	77	45	69	79	94	87	68
Country	63	77	75	64	59	63	43	58	56	44	56	60

**Supplemental immunisation coverage:** the country conducts four rounds of supplementary immunisation campaigns using polio vaccines yearly. All children under five of age are targeted and given two drops of the polio vaccines irrespective of the child’s polio vaccination status, which boosts polio herd immunity. Results are collated against set targets, with an evaluation done to determine the coverage and quality of the campaign. The country has conducted over 32 OPV campaigns since 2011. Table 2 shows the administrative data obtained directly from the vaccinators’ record and post-campaign evaluation (PCE); data obtained from independent assessors was conducted two days after the nationwide polio campaigns. The administrative results ranged from 77% to 111%, while the PCE results obtained from independent monitors ranged from 81% to 97%. Table 2 shows that the number of rounds declined from four in 2011 to two in 2019 and none in 2020. The last polio countrywide supplemental immunisation activities (SIAs) were conducted in 2019 and achieved a coverage rate of 100%; however, post-campaign evaluation (PCE) showed coverage of 92%. However, in rounds 4, 2016 and round 1, 2018, the PCE was higher than administrative data. Table 3 shows the administrative and PCE results by state, with Upper Nile recording the lowest admin coverage of 22% and Central Equatoria with the highest admin coverage of 117%. Table 3 also showed that post-campaign evaluation was not conducted for some of the conflicts affected states rounds even though an SIA was done.

**Table 2:** comparison of administrative and post campaign evaluation results of supplemental immunisation activities conducted 2011 - 2020

Year	Round I (%)		Round II (%)		Round III (%)		Round IV (%)	
	Admin	PCE	Admin	PCE	Admin	PCE	Admin	PCE
2011	95	90	94	90	96	94	96	96
2012	102	95	102	94	100	93	105	94
2013	105	95	104	95	105	90	82	NA
2014	94	81	110	93	100	89	108	89
2015	112	94	111	94	110	94	111	95
2016	118	97	95	94	92	88	87	90
2017	90	88	90	89	91	86	90	88
2018	77	84	96	87	NA	NA	93	85
2019	98	89	97	92	NA	NA	NA	NA
2020	NA	NA	NA	NA	NA	NA	NA	NA

* 2020 no NID done due to the COVID 19 pandemic and lockdown; * Admin refers to administrative coverage result of the polio campaigns; * PCE refers to post campaign evaluation result of the polio campaigns; * NA refers to not applicable

**Table 3:** results of polio campaigns 2013-2019, South Sudan

	2013 (Apr 2013)		2014 (Apr 2014)		2015 (Mar 2015)		2016 (Apr 2016)		2017 (Mar 2017)		2018 (Apr 2018)		2019 (Apr 2019)	
Name of States	Admin %	PCE %	Admin %	PCE %	Admin %	PCE %	Admin %	PCE %	Admin %	PCE %	Admin %	PCE %	Admin %	PCE %
Central Equatoria	115	95	115	76	114	99	117	86	67	87	79	87	85	93
Eastern Equatoria	116	93	102	70	116	91	107	92	106	86	102	93	101	86
Jonglei	95	96	ND	ND	ND	ND	82	ND	91	ND	86	84	99	90
Lakes	99	96	103	84	116	98	127	99	119	93	128	88	123	90
Northern Bahir Ghazal	100	92	68	72	101	92	96	97	111	93	113	92	113	93
Unity	97	96	ND	ND	ND	ND	97	ND	84	ND	114	85	109	91
Upper Nile	106	98	ND	ND	112	ND	58	ND	22	ND	38	81	33	98
Warrap	106	94	75	86	111	89	99	100	114	94	113	85	111	95
Western Bahir Ghazal	105	91	67	86	111	96	104	95	83	ND	97	91	102	88
Western Equatoria	97	97	149	86	103	94	69	89	97	86	98	86	100	88
**South Sudan**	**104**	**95**	**93**	**81**	**111**	**94**	**95**	**94**	**90**	**89**	**96**	**87**	**96**	**92**

* Admin = Administartive coverage; **PCE = Post campaign evaluation; ***ND = Not done

**Figure 3:**
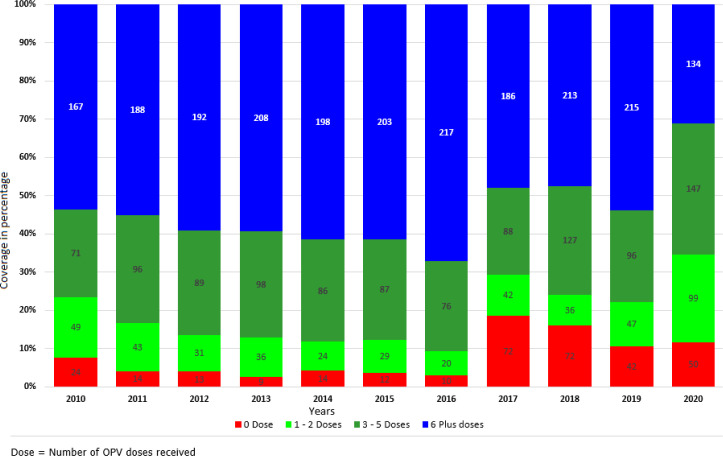
immunity profile for non-polio - AFP cases (6-59 months) 2010-2020 South Sudan

**Immunity profile for non-polio AFP cases:** a dataset of AFP surveillance comprised 3970 AFP cases notified from 2010 to 2020, of which 3161 (80%) of the cases were aged 0 to 59 months. Overall, 3182 (80%) of notified AFP cases had received more than three doses of the oral polio vaccine, and the polio vaccination status was zero for 332 (8%) of the AFP cases (Figure 3). The proportion of AFP cases with more than three OPV doses varied between 65% and 91% between 2010 to 2020, with the lowest rates observed in 2020 (65%), while the highest percentage was observed in 2016 (91%).

**Figure 4:**
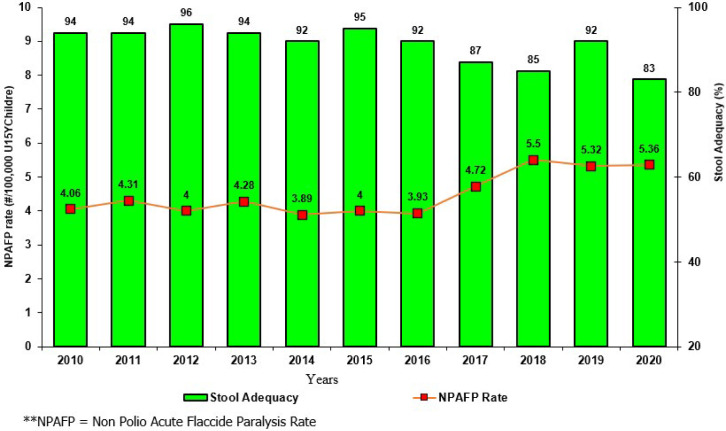
the two main acute flaccid paralysis surveillance indicators 2010-2020, South Sudan

**AFP surveillance indicators:** from 2010 to 2020, South Sudan maintained the two main indicators of AFP surveillance, well above recommended standard, with the NP-AFP rate above 3/100,000 children under 15 years of age and reaching an NP-AFP rate of 5.36/100,000 for 2020, with the NP-AFP ranging from 3.89 to 5.5, with the highest number reported in 2018 (Figure 4). Likewise, stool adequacy has been above the certification level (80%) since 2010 and was 83% for 2020, with the best performance recorded in 2019. The URVI laboratory reports above the recommended standard of 10% NPENT since 2010, with the highest NPENT rate of 21.5% recorded in 2019 and the lowest NPENT rate of 13.2% recorded in 2018 (Table 4).

**Table 4:** non-polio enterovirus rate of acute flaccid paralysis samples 2010-2020, South Sudan

Year	Number of acute flaccid paralysis cases	Number of cases with enteroviruses	Non- polio enterovirus rate
2010	309	43	13.9%
2011	341	56	16.4%
2012	324	66	20.4%
2013	350	74	21.1%
2014	318	51	16.0%
2015	331	60	18.3%
2016	323	67	20.7%
2017	388	68	17.5%
2018	447	59	13.2%
2019	400	86	21.5%
2020	429	79	18.4%

## Discussion

South Sudan’s polio eradication program has seen its share of successes and setbacks, with routine immunisation suffering the most. Two vaccines, the OPV and the IPV protect against wild poliovirus and are both routinely administered in the country [29,30]. The outcomes of this study indicated that routine immunisation coverage continues to drop and is significantly below the required coverage of OPV3 and IPV of 80%. WUENIC reports and two independent EPI coverage surveys undertaken in 2012 and 2017 corroborate this conclusion [31,32]. The consistent low coverage between 2015 and 2020 can be attributed primarily to the conflict. This has weakened the health system, particularly at the peripheral level, with far-reaching negative consequences for engaging, retaining, and motivating adequate skilled health workers to deliver quality immunisation services [33,34]. However, the severe dips in 2014 and 2016 are explained by the massive conflict in 2013. Similar findings have been reported in conflict-affected nations such as the Syrian Arab Republic and Ukraine, with vaccination coverage declining by 50% [35].

Many health services in the country are provided in collaboration with partner organisations. However, in 2019, there was a delay in contract signing between the MOH and these partners, affecting many health services for nearly six months, as evidenced by the lowest coverage ever reported in the country that year. The three-former conflict-affected states of Jonglei, Unity and Upper Nile bore the major conflict’s brunt. They continuously reported the lowest routine immunisation coverage, with most health facilities vandalised. There are also challenges in the vaccine supply chain, depleted human resources, and high population displacement and movement, with none of their counties reporting coverage above 80%. The increased coverage of >100% in Central Equatoria and Warrap states in 2013 and 2014 can be attributed to the massive population movement to safer havens. The disparity between administrator coverage, the EPI survey, and WUENIC demonstrates that data quality is a concern. The country is now conducting training sessions and quarterly data quality assessments to identify and close this gap. The country’s general poor coverage is comparable to that of several other nations involved in the conflict, including Pakistan, Afghanistan, Yemen, Somalia, and the Democratic Republic of the Congo [36,37]

It is critical to recognise that the three states of Unity, Jonglei, and Upper Nile will require a more tailored strategy to increase RI coverage and mitigate the danger of polio epidemics. These states have suffered the most from the conflict, as these were where the fighting was most intense as facilities were burnt and people displaced with its attendant poor RI coverage. The country undertakes Supplemental Immunisation Activities (SIAs) through National Immunisation Days (NIDs), each cycle targeting about 3 million children aged <5 years to supplement the herd immunity produced by routine vaccination. These campaigns occur 4 times a year and are conducted door-to-door with vaccinators stationed at vehicle parks, schools, and border crossings to guarantee no child is missed. This approach has been highly successful, with coverage rates >85% commonly reported and verified by third-party post-campaign evaluations. It is worth noting that NIDs remain the only source of immunisation services in many sections of the country. This is supported by an analysis of children <5 years of age reporting AFP, which indicates that, on average, 80% of AFP cases received three or more doses during the polio campaigns from 2010 to 2020. Population immunity to polio may be higher than reported administrative coverage due to the numerous polio campaigns conducted.

Only three campaigns have been conducted in the last three years, two of which were subnational immunisation days targeting states with low RI administrative coverage. This has led to a reduction in the number of < 5-year-old children from AFP cases receiving three or more doses of OPV vaccine. This figure was lowest in 2020, with only 65% of reported AFP cases receiving three or more OPV doses due to the absence of polio campaigns which were halted due to the COVID 19 pandemic [38]. Because of campaign reductions, the decline in herd immunity will make the country vulnerable and or susceptible to another WPV importation, as reported in 2006 and 2008. Administrative results for the campaigns show that there is not much difference in coverage between the states, except for Upper Nile which consistently reported low coverage due mainly to access and insecurity. At the peak of the conflicts from 2014 to 2016, nationwide polio campaigns were not held for the former conflict states of Jonglei, Upper Nile and Unity as access was impossible due to insecurity; however other tailored approaches such as subnational campaigns and hit and run were conducted. The campaign’s administrative results can be attributed to the quality of the pre-implementation activities, including micro plans, adherence to team selection criteria when recruiting team members, appropriate social messages with minimal non-compliance, and collaboration with partners led by the MOH. Also, the use of innovative tools such as the ODK for reporting performance and, most importantly, institutional memory, as the country has extensive experience in conducting similar campaigns. The high coverage in polio campaigns has proven challenging to transfer to RI, and the same has been recorded in other conflict-affected countries such as Somalia and warrants additional investigations [39].

South Sudan has consistently met the two core AFP surveillance indicators between 2010 and 2020, with improvements noted in the last three years attributed to the scaling-up of the community surveillance system in hard-to-reach states by the CORE group and access for humanity. Innovations such as the Open data kit, which uses mobile phones to provide real-time data for action and serve as the foundation for the programme’s accountability framework [6]. The country reported 12 wild poliovirus outbreaks in 2004-2005 and 64 in 2008-2009. It reported 2 and 50 circulating Vaccine-Derived Polio Virus (cVDPV) outbreaks in 2014 and 2020, respectively, demonstrating a sound surveillance system while pointing to low herd immunity against polio virus.

South Sudan’s AFP surveillance indicators are higher than the regional average for East and Southern African countries, with an NP-AFP at 5.1 and stool adequacy at 90% compared to the region’s NP-AFP at 3.3 and stool adequacy at 86% [40]. This is due to the significant investment made by donors and the GPEI collaboration and the limited reliance on the current government health system, which is based on a passive surveillance system. Conflicts appear to have little effect on the ability to detect AFP cases and meet standard indicators, with similar results reported in countries such as Pakistan, Afghanistan, and Nigeria [39,41,42] The program’s ability to detect and report the presence or absence of poliovirus is contingent upon the laboratory’s ability to isolate and identify enteroviruses from AFP samples. These are affected by the reverse cold chain, with a 10% NPEV isolation rate serving as a reference point [43]. The country met the criterion for this indicator, further establishing the credibility of the country’s polio activities despite delays in stool transfer to WHO-accredited laboratories caused by erratic flight schedules and cancellations, particularly during the rainy season. Transportation of AFP samples to the URVL on time continues to be the most significant barrier to the country’s polio program. However, it can be addressed by ensuring that samples are adequately preserved until transported to the laboratory.

The findings of this study should be interpreted in the context of several limitations. First is the incompleteness and varied timeliness of the routine immunisation data. Second, the target population used remains data from the extrapolated 2008 Census. To overcome this, the RI data was the final database submitted in subsequent years, e.g. the 2020 data used was sourced from the DHIS database of March 2021. Also, data from the polio campaign micro plans collated from the community via a bottom-top process are used for campaign planning. For surveillance data, incomplete variables were excluded from any analyses; also, we conducted data harmonisation between different partners for consistency. The country routinely validates all AFP cases by senior and trained personnel. The introduction of the ODK in 2017 has helped mitigate some of the above limitations and the monthly data harmonisation at the National, state, and county levels. Second, the AFP cases reported through the polio surveillance system may not reflect the actual numbers of all AFP cases in the country. The major factors responsible are no reporting or investigation of these cases by the caregivers or health workers due to lack of knowledge and difficulty reporting due to poor or no mobile network. These are mitigated by yearly training of health workers and community informants. Third, the high NP-AFP rate does not imply that these are all true AFP cases, but this has been addressed by validating all AFP cases by senior WHO officers.

## Conclusion

South Sudan continues to report low immunisation coverage threatening polio achievements. Polio eradication is possible even in conflict settings with limited health service capabilities; however, post-certification guidelines set by GPEI must be followed to avoid a resurgence of the poliovirus. As GPEI comes to an end, it is essential to bring innovative ideas to improve routine immunisation coverage quickly. Of concern is the reduction in the number of polio campaigns which provides an opportunity for children to be immunised with OPV and an opportunity for sensitisation of caregivers on the need for immunisation.

**Recommendations:** we recommend that these polio campaigns be maintained with at least two national campaigns every year until RI coverage improves. Human resources are the most asset of the polio programme and need to be adequately supported as the GPEI ramps down with a decline in funding. The AFP surveillance system must be maintained post-certification, as the country’s IDSR moves from the passive mechanism to the active search done for AFP surveillance. As the country continues to build and improve the healthcare infrastructure, there is a need for continued AFP surveillance to detect and respond to poliovirus outbreaks Tailored strategies such as Periodic Intensified Routine Immunisation (PIRI), Enhanced mobile and outreach interventions, missed opportunities for vaccination need to be adopted for RI use in the country, and proper compensation and security provided for health workers.

### What are the implications for Public health?

Polio free certification is just the first of many steps towards achieving a polio-free world. Conflict countries like South Sudan still need to conduct high-quality polio campaigns, strengthen its AFP surveillance system for many more years as the current routine immunisation coverage will be unable to provide herd immunity required to stop the importation of the poliovirus in the country.

### What is known about this topic

- As of 2020, only one WHO region is yet to be certified wild poliovirus free, and the countries in this region (Afghanistan and Pakistan) along with South Sudan have suffered years of conflict; however, South Sudan has succeeded in getting its claim of being wild poliovirus free validated and accepted;
- The country continues to report low routine immunisation coverage, less than the expected 80% for all vaccines, including both OPV and IPV
- Injectable polio vaccine was introduced in 2016 following the switch from tOPV to bOPV.

### What this study adds

- It elaborates how WPV free certification can be achieved even in conflict countries and how the application of tested polio strategies can stop the virus on its track;
- The importance of polio campaigns in maintaining herd immunity;
- It identifies states and counties that need to be prioritised where resources are limited.
